# Plasma C-Terminal Agrin Fragment as an Early Biomarker for Sarcopenia: Results From the GenoFit Study

**DOI:** 10.1093/gerona/glab139

**Published:** 2021-05-16

**Authors:** Jedd Pratt, Giuseppe De Vito, Marco Narici, Ricardo Segurado, Ludmilla Pessanha, Jackie Dolan, Judith Conroy, Colin Boreham

**Affiliations:** 1 Institute for Sport and Health, University College Dublin, Ireland; 2 Genuity Science, Dublin, Ireland; 3 Department of Biomedical Sciences, CIR-Myo Myology Centre, Neuromuscular Physiology Laboratory, University of Padova, Italy; 4 Centre for Support and Training in Analysis and Research, and School of Public Health, Physiotherapy and Sports Sciences, University College Dublin, Ireland; 5 Conway Institute of Biomolecular and Biomedical Research, University College Dublin, Ireland

**Keywords:** Agrin, Muscle strength, Muscle wasting, Neuromuscular junction, Skeletal muscle mass

## Abstract

Barriers associated with direct muscle quantification have prevented a consistent implementation of therapeutic measures for sarcopenia. Recently, the relevance of circulating C-terminal agrin fragment (CAF) as an accessible screening method alternative for sarcopenia has gained credence. Accordingly, this study aimed to verify the pertinence of plasma CAF as a biomarker for sarcopenia. Three hundred healthy adults aged between 50 and 83 years took part in this study. Sarcopenia was diagnosed according to the European Working Group on Sarcopenia in Older People criteria. Body composition was assessed using dual-energy x-ray absorptiometry, while muscle strength was examined using hand dynamometry. Plasma CAF concentrations were determined using a commercially available ELISA kit. CAF concentrations were significantly associated with appendicular lean mass (ALM), but not grip strength (*p* = .028, *p* = .575, respectively). Plasma CAF concentrations were significantly elevated in sarcopenic individuals compared to nonsarcopenic (*p* < .001). Overall, individuals with low grip strength or low ALM displayed significantly higher CAF levels compared to healthy controls, after adjusting for age and body mass index (*p* = .027, *p* = .003, respectively). In males, those with low grip strength or low ALM had significantly elevated CAF levels (*p* = .039, *p* = .027, respectively), while in females, only those with low ALM had significantly raised CAF concentrations, compared to healthy controls (*p* = .035). Our findings illuminate the potential relevance of CAF as an accessible biomarker for skeletal muscle health. CAF determination may enhance clinical practice by facilitating more widespread treatment strategies for sarcopenia. Nevertheless, future research is needed to confirm the diagnostic pertinence of CAF concentrations in screening for sarcopenia.

Sarcopenia, characterized by an age-related loss of skeletal muscle mass, strength, and function (1), is a strong predictor of multiple adverse health outcomes such as physical disability (2), hospitalization (3), stroke (4), and mortality (5). With prevalence rising from 10% in individuals aged 60–69 years, to over 40% in adults over 80 years of age (6), sarcopenia represents, therefore, a major public health concern. Moreover, given that the prevalence of sarcopenia is expected to rise in line with societal aging, age-related muscle degradation will impose an increasing burden on health care systems globally. Therefore, the elucidation of effective management strategies should be a high priority.

Unfortunately, to date, diagnostic and treatment efforts have been greatly impeded by the lack of a consensus definition for sarcopenia. While early definitions were centered upon the presence of low muscle mass (1,7), in 2010, the European Working Group on Sarcopenia in Older People (EWGSOP) proposed a diagnostic algorithm incorporating both muscle mass, and function as fundamental components (8). Since then, various operational definitions have been suggested in an attempt to enhance the efficacy of the sarcopenic phenotype in predicting important clinical outcomes such as functional disability, falls, and mortality (9–11). Correspondingly, a growing degree of importance has been placed on muscle strength, while the clinical relevance of muscle mass, as originally thought, has been challenged. This shift is underscored by a series of recent papers by the Sarcopenia Definitions and Outcomes Consortium (SDOC) demonstrating grip strength to have powerful predictive capacity for mobility limitations and mortality, while illustrating a lack of transfer between muscle mass measurements and poor health outcomes (12–14). Nevertheless, presently, both muscle mass and function are 2 commonly used components in many definitions, while the necessity for one, consensus operational definition is ongoing.

Furthermore, although the criteria elaborated by groups such as the EWGSOP, Asian Working Group for Sarcopenia, Foundation for the National Institutes of Health, and SDOC guide the assessment of sarcopenia (9), difficulties surrounding the ascertainment of accurate muscle mass and function data further prevent a consistent implementation of diagnosis and treatment protocols. While techniques such as dual-energy x-ray absorptiometry, magnetic resonance imaging, computed tomography, and bioimpedance can be used to assess lean mass (15,16), such methods, with the exception of bioimpedance, can be both costly and of limited availability, particularly in community settings. Additionally, challenges associated with the collection of valid muscle function data from older populations can present further impediments to the assessment of sarcopenia. As a result, direct methods of muscle mass and muscle performance quantification are not always viable for the general population. However, the use of a blood-based biomarker may represent a cost-effective and accessible alternative screening method for sarcopenia. The elucidation of such a marker could facilitate widespread diagnostic and therapeutic measures, reducing the barriers associated with the measurement of muscle mass and performance, while attenuating the health and economic consequences of sarcopenia.

While the pathogenesis of sarcopenia is known to be multifactorial (17–19), the importance of neurophysiological processes in maintaining skeletal muscle health with advancing age has recently gained credence (20,21). In particular, a reduced reinnervative capacity evoked by age-related disruption at the neuromuscular junction (NMJ) is recognized as a key contributing factor to the development and progression of sarcopenia (22). Indeed, the NMJ, essential for nerve-to-muscle crosstalk, undergoes significant remodeling with advancing age, whereby the precise alignment between pre- and postsynaptic structures is reduced, leading to a progressive accumulation of denervated muscle fibers (20). Accordingly, biomarkers of NMJ stability have been proposed as early indicators of sarcopenia (23).

Agrin (AGRN), a synaptically located heparan sulphate proteoglycan, is a well-established mediator of NMJ formation and stabilization (24). The regulation of the molecular assembly of pre- and postsynaptic structures and the proper aggregation of acetylcholine receptors is largely orchestrated by AGRN-mediated signaling. As neuromuscular remodeling occurs, the neuronal protease neurotrypsin proteolytically cleaves and inactivates AGRN, dissociating a 22-kDa C-terminal agrin fragment (CAF) (25), easily quantifiable in human blood. Recently, circulating CAF concentrations have emerged as a potential biomarker of skeletal muscle deterioration (26), due to its inverse relationship with NMJ health.

Excessive cleavage of AGRN has been shown to evoke precocious sarcopenia (27). Thus, circulating levels of CAF may represent an early indicator of NMJ dismantling and muscle fiber denervation, which signals the onset of sarcopenia. Intriguingly, a recent bed-rest study found a significant rise in CAF concentrations and in the number of neural cell adhesive molecule positive fibers (marker of denervation) after only 10 days of bed rest (28), indicating the sensitivity of CAF as a marker of NMJ damage in the presence of muscle denervation. Moreover, there is growing evidence to support the viability of CAF as a biomarker for sarcopenia in a variety of subpopulations (23,26,29,30). Together, these findings suggest that CAF may be a useful diagnostic tool for sarcopenia while also demonstrating its potential as an early indicator of denervation. Hence, further illumination of its relevance could support a timely implementation of targeted therapeutic and preventative strategies, ultimately focused on attenuating the development of sarcopenia.

Accordingly, the aim of this study was to examine the indicative relevance of plasma CAF concentrations to sarcopenic phenotypes in a large sample of healthy older adults.

## Method

### Participant Characteristics

Participants were enrolled as part of the GenoFit study, a cross-sectional cohort study examining the link between genetics, health, and fitness, located in Dublin, Ireland. Three hundred participants aged between 50 and 83 years were randomly selected from a pool of 1728 individuals aged ≥50 years (males, *n* = 150; mean age: 64.2 ± 8.7 years and females, *n* = 150; mean age: 63.9 ± 8.3 years). To be eligible, participants had to be aged ≥50 years, be without renal function abnormalities, be free from any chronic disease or musculoskeletal injury that may affect muscle mass and/or muscle strength, and be able to provide informed consent. The study protocol was approved by the University College Dublin’s (UCD) Research Ethics Committee and written informed consent was obtained from all participants at enrollment.

### Body Composition Analysis

Dual-energy x-ray absorptiometry (Lunar Prodigy, GE Healthcare Technologies, Chicago, IL) was used to assess body composition. Appendicular lean mass (ALM) was determined as the combined lean mass of the limbs. All dual-energy x-ray absorptiometry scans were referred by a registered physician and performed in the Human Performance Laboratory in UCD by a trained technician.

### Muscle Strength Testing

Grip strength was assessed using a Jamar digital handheld dynamometer (JLW Instruments, Chicago, IL). Participants performed 2 attempts with each hand while in a standing position with their arm positioned straight by their side. The average value from the highest attempt from each hand was used for analysis.

### Sarcopenia Identification

Sarcopenia was identified in accordance with the EWGSOP2 criteria (9). As such, sarcopenia was diagnosed according to the presence of both low muscle strength and low muscle mass, while presarcopenia was based upon the presence of either low muscle strength or low muscle mass. Cutoff points were established using the sex-specific lowest quintile of muscle strength and muscle mass measurements from the study population. Accordingly, low grip strength was defined as <32.95 kg and <20.40 kg, for males and females, respectively, and low ALM was defined as <23.01 kg and <14.95 kg, for males and females, respectively.

### Blood Sampling and Plasma CAF Measurement

Blood samples were obtained by venepuncture of the median cubital vein using vacutainers containing an EDTA anticoagulant (BD Vacutainer). Samples were allowed to rest for 30 minutes prior to centrifugation. For plasma separation, samples were centrifuged at 4000*g* for 10 minutes at 4°C and the extracted plasma was stored in aliquots at −80°C until analysis. Plasma CAF concentrations was determined using a commercially available ELISA kit (Abcam #ab216945) according to the manufacturer’s instructions.

### Statistical Analysis

Results are presented as mean ± standard deviation (*SD*), unless stated otherwise. Independent-sample Student’s *t* test was used to assess differences between population characteristics according to the presence of sarcopenia. The association between ALM, grip strength, and age was examined using Pearson’s correlation coefficient. Multiple linear regression models were used to determine the association between CAF concentrations, ALM, and grip strength while adjusting for potential confounders such as sex, age, and body mass index (BMI). Analysis of covariance was performed with the same models to compare mean plasma CAF levels between sarcopenic domains, with age and BMI as covariates. The utility of plasma CAF for diagnosing sarcopenia, low ALM, and low grip strength was further examined using receiver operating characteristic (ROC) curve analysis. All statistical analyses were performed using SPSS software (Version 26, IBM SPSS Inc., Chicago, IL). Statistical significance was set at *p* < .05 for all tests, with no adjustment for multiple testing.

## Results

### Study Population

The main characteristics of the study population are displayed in Supplementary Table 1. In total, 300 participants took part in this study with an equal distribution of males and females (150 men and 150 women). Thirty-one participants were diagnosed with sarcopenia (10.3%) with no significant differences noted between gender (9.3% prevalence in men and 11.3% in women, *p* = .571). Compared to nonsarcopenic controls, sarcopenic individuals had significantly elevated plasma CAF concentrations (*p* < .001) and lower BMI, body mass, and height (*p* < .001, <.001, and .011, respectively).

### ALM, Grip Strength, and Age

**Figure 1.**
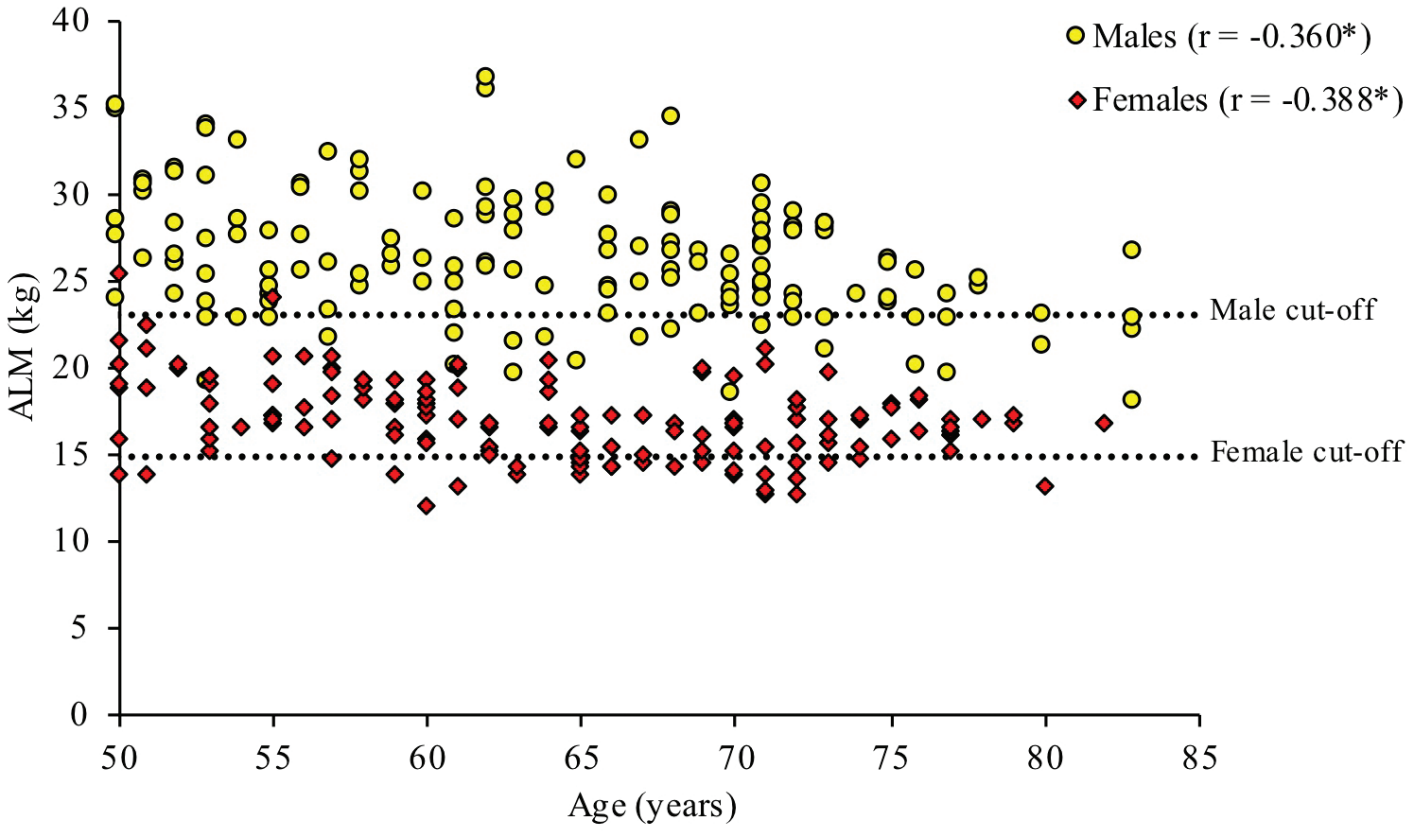
Association between appendicular lean mass (ALM) and age, stratified by sex. Males: *N* = 150, cutoff for low ALM = 23.01 kg; females: *N* = 150, cutoff for low ALM = 14.95 kg; *p < .001.

**Figure 2.**
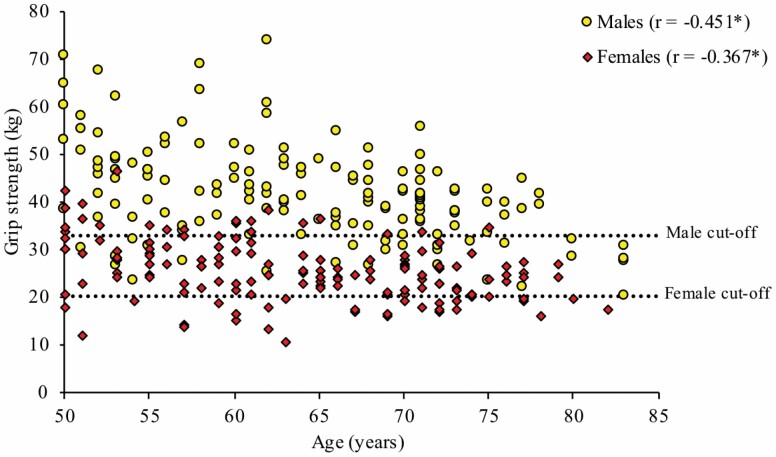
Association between grip strength and age, stratified by sex. Males: *N* = 150, cutoff for low grip strength = 32.95 kg; females: *N* = 150, cutoff for low grip strength = 20.40 kg; *p < .001.

Figures 1 and 2 illustrate the association between ALM, grip strength, and age for both sexes. For males and females, there were significant negative correlations between ALM and age (*r* = −0.360, *p* < .001 and *r* = −0.388, *p* < .001, respectively) and grip strength and age (*r* = −0.451, *p* < .001 and *r* = −0.367, *p* < .001, respectively). Interestingly, many individuals fell below the cutoff thresholds relatively early (~55 years of age), particularly for grip strength.

### CAF, Sarcopenia, and Presarcopenia

Supplementary Table 2 displays multiple regression models assessing the association between ALM, grip strength, and CAF concentrations. Levels of CAF were significantly associated with ALM when controlling for multiple potential confounders such as sex, age, and BMI (*p* = .028). Interestingly, such association was not observed between grip strength and CAF (*p* = .575). Analysis of covariance revealed CAF concentrations to be significantly higher among sarcopenic individuals compared to nonsarcopenic (*p* < .001) (Table 1). This association remained significant for both sexes after adjusting for age and BMI (*p* < .001 and *p* = .035 for men and women, respectively).

**Table 1.** Unadjusted and Adjusted Association Between Plasma CAF and Sarcopenia

	Sarcopenia (*N* = 31)	No Sarcopenia (*N* = 269)	*p* Value	Sarcopenia (*N* = 31)	No Sarcopenia (*N* = 269)	*p* Value
	Unadjusted Means (*SEM*)			Adjusted Means (*SEM*)*		
CAF (ng/mL)						
All participants	3.13 (0.24)	2.65 (0.03)	<.001	3.19 (0.12)	2.65 (0.04)	<.001
Men	3.38 (0.47)	2.63 (0.04)	<.001	3.42 (0.19)	2.62 (0.06)	<.001
Women	2.92 (0.22)	2.68 (0.05)	.109	2.99 (0.14)	2.67 (0.05)	.035

*Notes*: CAF = C-terminal agrin fragment; *SEM* = standard error of mean.

*Adjusted for age and body mass index.

Plasma levels of CAF were also significantly associated with presarcopenia (presence of low grip strength or low ALM). Indeed, across the entire sample, plasma CAF concentrations were significantly higher in individuals with low grip strength (*p* = .027) (Table 2) or low ALM (*p* = .003) (Table 3), when compared to those with normal grip strength or ALM. In the male population, individuals with low grip strength (*p* = .039) or low ALM (*p* = .027) had significantly elevated CAF levels when compared to healthy controls, after adjusting for age and BMI. In females, those with low ALM had significantly elevated CAF levels (*p* = .035) compared to those with normal ALM also when adjusted for age and BMI. Finally, while not statistically significant, a trend for higher CAF concentrations in females with low grip strength was observed (*p* = .391).

**Table 2.** Unadjusted and Adjusted Association Between Plasma CAF and Grip Strength

	Low Grip (*N* = 60)	Normal Grip (*N* = 240)	*p* Value	Low Grip (*N* = 60)	Normal Grip (*N* = 240)	*p* Value
	Unadjusted Means (*SEM*)			Adjusted Means (*SEM*)*		
CAF (ng/mL)						
All participants	2.93 (0.14)	2.65 (0.03)	.003	2.87 (0.08)	2.66 (0.04)	.027
Men	3.05 (0.23)	2.61 (0.05)	.004	2.95 (0.13)	2.64 (0.06)	.039
Women	2.82 (0.14)	2.68 (0.05)	.231	2.79 (0.11)	2.69 (0.05)	.391

*Notes*: CAF = C-terminal agrin fragment; *SEM* = standard error of mean.

*Adjusted for age and body mass index.

**Table 3.** Unadjusted and Adjusted Association Between Plasma CAF and ALM

	Low ALM (*N* = 60)	Normal ALM (*N* = 240)	*p* Value	Low ALM (*N* = 60)	Normal ALM (*N* = 240)	*p* Value
	Unadjusted Means (*SEM*)			Adjusted Means (*SEM*)*		
CAF (ng/mL)						
All participants	2.91 (0.14)	2.65 (0.03)	.006	2.94 (0.09)	2.64 (0.04)	.003
Men	2.96 (0.24)	2.63 (0.04)	.029	2.98 (0.14)	2.63 (0.07)	.027
Women	2.86 (0.15)	2.67 (0.04)	.105	2.92 (0.11)	2.66 (0.05)	.035

*Notes*: ALM = appendicular lean mass; CAF = C-terminal agrin fragment; *SEM* = standard error of mean.

*Adjusted for age and body mass index.

### ROC Analysis

The ROC curves for sarcopenia, low ALM, and low grip strength are presented in Supplementary Figure 1. The area under the curves (AUCs) for sarcopenia, low ALM, and low grip strength were 0.575 (95% CI, 0.451–0.699, *p* = .171), 0.536 (95% CI, 0.446–627, *p* = .382), and 0.559 (95% CI, 0.472–0.645, *p* = .160), respectively. The sensitivity and specificity were 61.3% and 51.7% for sarcopenia, 43.3% and 70% for low ALM, and 56.7% and 52.1% for low grip strength.

## Discussion

Although sarcopenia has profound health and economic consequences for patients and health care systems globally, the barriers associated with skeletal muscle testing have so far prevented a consistent implementation of diagnosis and treatment protocols. As a result, the potential role of blood-based biomarkers in providing an accessible, alternative screening method has recently gained credence. While the etiology of sarcopenia is complex, disruption at the NMJ has been recognized as a key contributor to muscle degradation (21). As previously described, AGRN performs an indispensable role at the NMJ, orchestrating the alignment of pre- and postsynaptic apparatus and proper acetylcholine receptor clustering (24). Cleavage, and the subsequent inactivation of AGRN by neurotrypsin, leads to the release of a 22-kDa CAF (25), easily measured in human plasma. As such, promising evidence has emerged surrounding the viability of circulating CAF as a diagnostic tool for sarcopenia. Accordingly, this study aimed to verify the relevance of plasma CAF concentrations as a biomarker for sarcopenia in a large sample of older adults.

The main results from this study indicate that: (i) plasma CAF levels are significantly elevated in sarcopenic and presarcopenic individuals (low grip strength or low ALM) compared to healthy controls; (ii) sex modifies the strength of relationship between CAF levels and sarcopenic domains, with a stronger association observed in the male population; (iii) CAF concentrations appear to be a stronger indicator of muscle wasting than muscle strength deterioration in healthy older adults.

Our findings confirm the association between plasma CAF levels and sarcopenia as previously observed (23,26,29,30), and confirm CAF as a biomarker for presarcopenic phenotypes. Indeed, building on promising current evidence, our study highlights the association of plasma CAF not only with sarcopenia status, but also with each presarcopenic domain. This finding is of particular importance as it illustrates the potential of CAF in detecting clinically meaningful stages of performance. Moreover, these findings were observed in a population with notably healthier skeletal muscle than previous reports, further exemplifying the pertinence of CAF in screening for sarcopenia.

However, the strength of associations between CAF and sarcopenic phenotypes is likely to be sex-specific. Indeed, our findings suggest that CAF may be a stronger marker of muscle degradation for males than females. Males with low ALM or low grip strength displayed significantly elevated CAF concentrations compared to healthy controls, while in females, only those with low ALM had significantly higher CAF levels. Moreover, while statistically significant, the strength of associations between CAF and sarcopenia, and CAF and ALM were considerably lower in females compared to males (*p* = .035 vs *p* < .001 and *p* = .035 vs *p* = .027, respectively). Such results are consistent with previous studies reporting associations between CAF levels and parameters of muscle mass and function in males, but not females (29,31,32). Together, these findings postulate sex as a likely mediator toward the precision of CAF as a biomarker for sarcopenia. While there remains a current paucity of evidence surrounding sex-specific differences in CAF associations, AGRN may have greater relevance in processes of muscle degradation in males, compared to females, where the complexity of the hormonal network may contribute toward a more multifactorial pathogenesis of sarcopenia. Interestingly, we found a stronger correlation between grip strength and age in males than females (*r* = −0.451 vs *r* = −0.367), while no such sex-specific difference was observed between ALM and age (*r* = −0.360 vs *r* = −0.388). This finding suggests that the regulation of muscle strength during aging may be particularly complex in females, which may help explain the nonsignificant associations we observed between CAF levels and muscle strength among females. While differences in muscle architecture and mechanisms of adaptation are well-established between sexes (33,34), knowledge surrounding such disparities in biomarkers remains elusive. Future exploration of the potential differences in CAF associations between sexes may further illuminate the pertinence of CAF as a marker of muscle deterioration.

In addition to the uncertainty surrounding sex-based differences in CAF associations, evidence relating to the relevance of CAF as a biomarker for muscle strength is inconsistent. Indeed, while significantly elevated CAF concentrations were demonstrated across each sarcopenic domain, our results suggest CAF may be a stronger indicator of muscle wasting than muscle strength deterioration, particularly in females. These findings are in accordance with previous reports demonstrating a relationship between circulating CAF and muscle wasting, but not physical function or muscle strength (29,35). Nonetheless, there is also promising evidence supporting the indicative pertinence of circulating CAF across every sarcopenic phenotype (26,32,36,37). It is likely that differences in population characteristics contribute toward such disparities. For example, our participants were healthy older adults aged 50–83 years, free from any chronic disease or musculoskeletal disorder, while in studies that reported CAF associations across each sarcopenia domain, the populations were prefrailty older adults (≥80 years of age) (26), chronic obstructive pulmonary disease patients (37), stroke patients (36), or patients with chronic heart failure (32). These participants are likely to be at a more advanced stage of muscle degradation compared with our sample. As such, we propose that CAF is primarily a marker for muscle wasting in healthy older adults and the indicative relevance of CAF toward muscle strength becomes more pronounced in later stages of muscle deterioration.

Intriguingly, a recent paper by Carson (38) proposed that while baseline grip performance is affected by skeletal muscle morphology, the age-related decline in grip strength is largely mediated by the integrity of neural systems, particularly in older adulthood. While upon initial consideration, it seems surprising, therefore, that circulating CAF, a marker of NMJ stability, was a stronger indicator of ALM, than grip strength in this study. However, it is important to note that the neural contribution toward grip performance is believed to increase with advancing age, as the contribution of skeletal muscle morphology weakens (38). Therefore, the greater importance of neural mechanisms in later adulthood may help explain why, in our study population, where skeletal muscle characteristics were relatively healthy, CAF was a stronger predictor of muscle mass, than strength. Indeed, such belief would support our postulation that the indicative pertinence of CAF toward muscle strength increases at more progressive stages of muscle decline. In that regard, future studies involving healthy, older participants may provide further validation toward the diagnostic relevance of circulating CAF to the general population.

Interestingly, in this study, ROC analysis revealed relatively low AUCs for sarcopenia, low ALM, and low grip strength (0.575, 0.536, and 0.559, respectively) while sensitivity and specificity ranged from 43.3% to 61.3% and 51.7% to 70%, respectively. These findings are in accordance with a recent study that found, when assessed individually, interleukin-6, secreted protein acidic and rich in cysteine, macrophage migration inhibitory factor, and insulin-like growth factor 1 displayed poorer diagnostic performance for sarcopenia compared to a combination model (AUC < 0.7 vs 0.763) (39). These findings reflect the complex etiology of sarcopenia, whereby a diverse network of pathways is likely to contribute toward skeletal muscle degradation (17–19). With that in mind, combining biomarkers into a single risk score that more holistically represents biological pathways may provide greater diagnostic accuracy for sarcopenia.

Additionally, it is worth mentioning that the relatively healthy population included in this study may help explain the low AUCs. Indeed, it is plausible that the diagnostic relevance of CAF increases at later stages of skeletal muscle deterioration. Therefore, future studies incorporating a sample at a more pronounced stage of skeletal muscle decline may provide further insight into the diagnostic power of CAF.

There are some limitations to this study. While grip strength is a well-established measure of overall muscle strength, it may also have been beneficial to assess lower limb strength, a useful indicator of functional ability in older populations (40). The inclusion of a parameter of muscle function, such as gait speed, may have provided further insight into the relevance of CAF concentrations toward muscle performance. Furthermore, while the presence of renal dysfunction was excluded from this study through self-reporting, a direct measurement of kidney function would have further strengthened our findings. Finally, while AGRN was the primary focus of this study, the potential benefits of combination models in increasing the sensitivity of sarcopenia diagnosis should be considered. As mentioned, a recent study found that combining interleukin-6, secreted protein acidic and rich in cysteine, macrophage migration inhibitory factor, and insulin-like growth factor 1 measurements into a single risk score enhanced the diagnostic accuracy compared to single biomarkers (39). Interestingly, AGRN was not included in the biomarker tests in this paper. In that regard, the diagnostic accuracy of combination biomarker models may be further enhanced with the addition of AGRN. Finally, while not a limitation of the study itself, it is important to note that the utility of CAF, or rather, any diagnostic biomarker is fundamentally reliant on the operational definition for sarcopenia. In that regard, while circulating CAF has promising screening utility for ALM and grip strength, there is an urgent need to establish a consensus definition for sarcopenia. Doing so, would not only aid with the clinical implementation of treatment and preventative protocols, but also enable a more targeted exploration into diagnostic biomarkers.

In conclusion, our findings demonstrate the potential of circulating CAF as an accessible indicator of skeletal muscle health in older adults. In that regard, CAF determination may help alleviate the need for exact muscle quantification and further enhance standard medical practice by facilitating a more widespread assessment of sarcopenia. Currently, CAF is the most promising biomarker for skeletal muscle health, with translational potential in facilitating timely interventions aimed at attenuating age-related muscle degradation. It is worth noting, however, that additional studies incorporating large samples more representative of the general population are needed to confirm the diagnostic relevance of CAF concentrations in screening for sarcopenia. Furthermore, future research should explore whether CAF levels may be a more useful biomarker for muscle degradation in males compared to females.

## Funding

This work was supported by the Irish Research Council (EBPPG/2019/9) to J.P.

## Conflict of Interest

None declared.

## Author Contributions

Conceptualization: J.P., G.D.V., and C.B.; data collection: J.P., G.D.V., C.B., and J.D.; data analysis: J.P., L.P., and R.S.; writing-original draft preparation: J.P.; writing-review and editing: J.P., G.D.V., M.N., C.B., R.S., J.D., J.C., and L.P.

## Supplementary Material

glab139_suppl_Supplementary_Materials
