# Supplementary material for: Plasma C-Terminal Agrin Fragment as an Early Biomarker for Sarcopenia: Results From the GenoFit Study
Source: J Gerontol A Biol Sci Med Sci. 2021 May 16;76(12):2090–6. doi: 10.1093/gerona/glab139 (PMC8599080; doi:10.1093/gerona/glab139)
Supplement: glab139_suppl_Supplementary_Materials [file glab139_suppl_supplementary_materials.pdf]

ONLINE-ONLY MATERIAL

eTable 1. Participant characteristics according to the presence of sarcopenia

	Sarcopenia (N=31)	No Sarcopenia (N=269)	P-value
Sociodemographic characteristics			
Age (years)	66.4 ± 10.4	63.8 ± 8.2	0.110
Sex			
Male, n (%)	14 (9.3)	136 (90.7)	0.571
Female, n (%)	17 (11.3)	133 (88.7)	
Anthropometric characteristics			
Height (cm)	164.3 ± 9.6	169 ± 9.6	0.011
Body mass (kg)	63.9 ± 11	75.8 ± 13.6	<0.001
BMI (kg/m²)	23.6 ± 3.4	26.4 ± 3.5	<0.001
Plasma biomarkers			
CAF (ng/ml)	3.1 ± 1.4	2.7 ± 0.5	<0.001
Sarcopenic phenotypes			
ALM (kg)	17.7 ± 4	22.1 ± 5.4	<0.001
Grip strength (kg)	22.5 ± 6.5	34.9 ± 11.4	<0.001

BMI = body mass index; CAF = C-terminal agrin fragment; ALM = Appendicular lean mass

eTable 2. Multiple regression examining the association between: 1) ALM and CAF; and 2) Grip strength and CAF

Variables	Unstandardised coefficients	95% CI	P-value	R^2
ALM [a]				
CAF	-0.495	-0.935 - -0.055	0.028	0.801
Grip strength [a]				
CAF	-0.394	-1.774 - 0.986	0.575	0.564

[a] = adjusted for sex, age and body mass index; N = 300; Dependent variables: model 1 = ALM, model 2 = Grip strength; Adjusted R^2: model 1 = 0.798, model 2 = 0.558; ALM = Appendicular lean mass; CAF = C-terminal agrin fragment

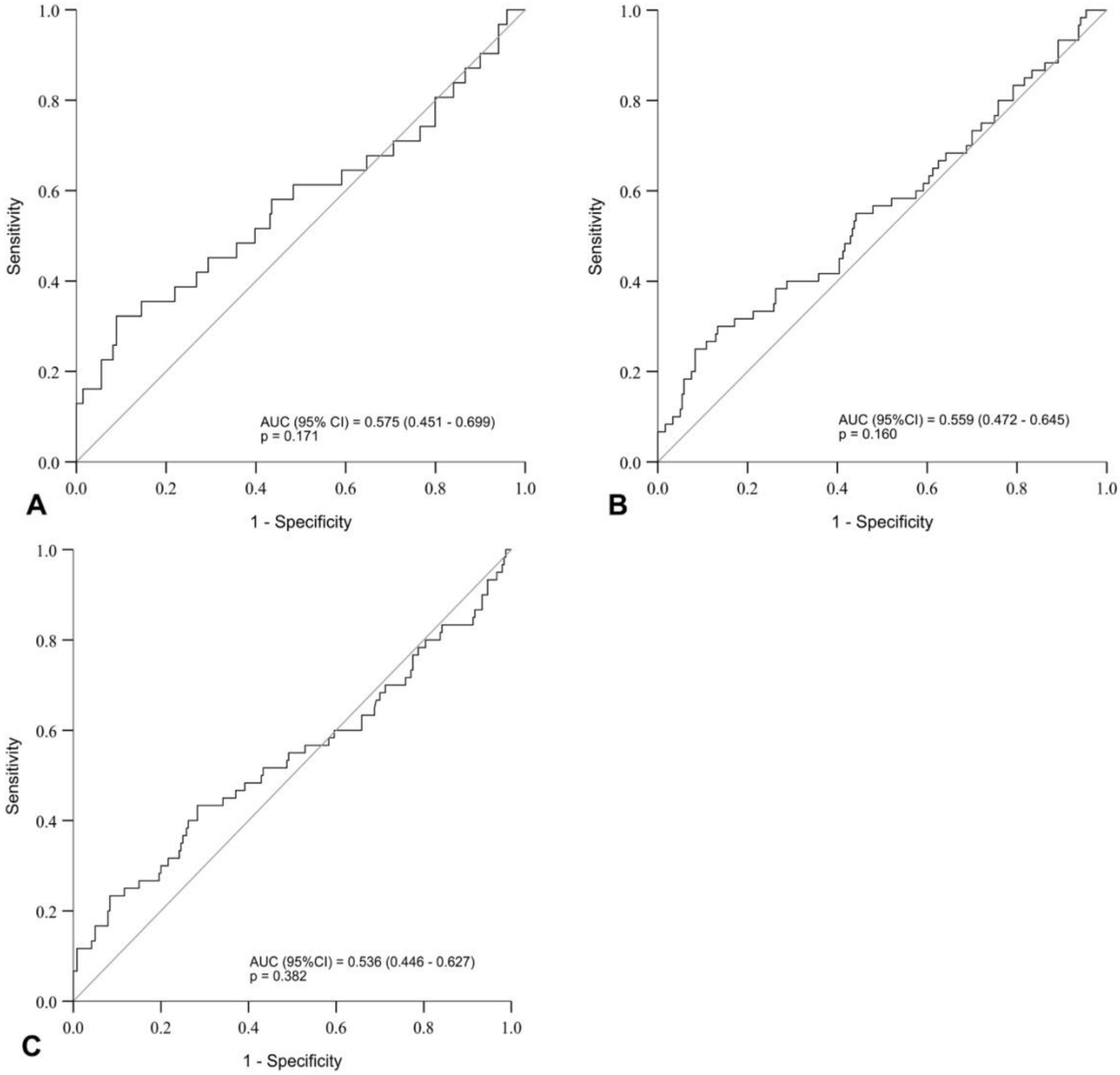

eFigure 1. Receiver operating characteristic analysis of C-terminal agrin fragment for: A) Sarcopenia; B) Low grip strength; and C) Low appendicular lean mass
